# Egg‐Inspection Frequency Predicts Egg Recognition but Not Ejection in a Cuckoo Host

**DOI:** 10.1002/ece3.73595

**Published:** 2026-05-20

**Authors:** Guobin Zheng, Jinggang Zhang, Zixuan Lin, Lixing Yang, Jianqiang Li, De Chen, Donglai Li, Wenhong Deng

**Affiliations:** ^1^ Ministry of Education Key Laboratory for Biodiversity Sciences and Ecological Engineering, College of Life Sciences Beijing Normal University Beijing China; ^2^ Provincial Key Laboratory of Animal Resource and Epidemic Disease Prevention, College of Life Sciences Liaoning University Shenyang China; ^3^ Department of Biology, Faculty of Arts and Sciences Beijing Normal University Zhuhai China; ^4^ School of Ecology and Nature Conservation Beijing Forestry University Beijing China

**Keywords:** cuckoo parasitism, Daurian redstart, egg ejection, egg inspection, egg recognition

## Abstract

One of the most effective strategies for avian hosts to defend against brood parasitism is to reject the parasitic egg from the focal nest. However, substantial inter‐individual variability in egg‐rejection behavior remains unexplained. A critical prerequisite for egg ejection is the recognition of the parasitic egg. Hosts may improve recognition accuracy by increasing inspection frequency and duration, thereby facilitating comparisons between the foreign egg and their own eggs. In this study, we investigated the relationship between egg inspection and egg‐rejection behavior in the Daurian redstarts *Phoenicurus auroreus* , a common host of the common cuckoo *Cuculus canorus* . Following the introduction of a model cuckoo egg, redstart females increased both the frequency and duration of egg inspection. Moreover, individuals that inspected the parasitic egg more frequently and for longer were more likely to recognize it. However, inspection behavior was not associated with subsequent egg ejection. Therefore, our results demonstrate that egg inspection enhances recognition of the parasitic egg but does not directly translate into ejection decisions.

## Graphical abstract

Redstarts inspected the foreign egg more frequently than they inspected their own eggs. Redstarts spent a longer time inspecting the foreign egg than viewing their own eggs. Individuals inspecting the foreign egg more often were more likely to recognize it.

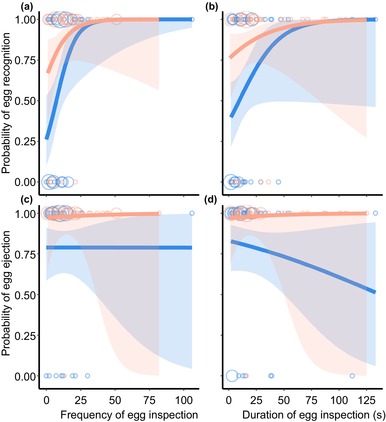

## 1 Introduction

Avian brood parasites lay their eggs in the nests of other bird species, imposing high costs on hosts (Davies 2000). To counter this, hosts have evolved various adaptations, among which egg rejection is one of the most common and effective strategies (Davies and Welbergen 2008; Gill and Sealy 2004; Medina et al. 2020; Rands 2012; Shen et al. 2021). However, considerable variation exists in the probability of egg rejection both within and among host populations (Davies and Brooke 1989; Liang et al. 2016; Medina et al. 2020; Moksnes et al. 1991), and understanding this variation remains challenging.

Many factors have been identified as influencing hosts' egg‐rejection behavior. First, in some species, such as gray catbirds *Dumetella carolinensis* (Kuehn et al. 2014) and American robins *Turdus migratorius* , egg rejection is genetically determined (Peer et al. 2011). Second, the degree of similarity between the foreign egg and host eggs can strongly influence rejection decisions (Giovanni et al. 2023; Honza and Cherry 2017; Li et al. 2020; Samaš et al. 2021; Yang et al. 2010; Ye et al. 2023). Third, egg rejection can be affected by parasitism risk, with hosts being more likely to reject the foreign egg under high‐risk conditions (Davies and Brooke 1988; Lindholm and Thomas 2000). Fourth, physiological regulation may also play a role; for example, hormones such as corticosterone (Abolins‐Abols and Hauber 2020; Turner et al. 2022) and prolactin (Ruiz‐Raya et al. 2021) have been shown to influence egg‐rejection behavior. In addition, nest luminosity (i.e., visibility inside the nest) is a crucial factor in egg rejection, as increased light level in the nest can enhance the recognition of the foreign egg (Honza et al. 2014; Langmore et al. 2005). Finally, individual personality may also egg rejection. For instance, bold individuals are more likely to reject the parasitic egg in Daurian redstarts *Phoenicurus auroreus* (Zhang, Møller, et al. 2021). Therefore, a substantial proportion of inter‐individual variation in egg rejection remains unexplained.

Moreover, egg rejection is a complex process that involves multiple stages, including egg recognition, decision‐making, and egg ejection (Hauber and Sherman 2001; Ruiz‐Raya and Soler 2017). Host ability to recognize the parasitic egg is often inferred from rejection rates (Davies and Brooke 1989; Marchetti 2000; Moskát et al. 2002; Spottiswoode and Stevens 2011; Thorogood and Davies 2013). The absence of a response to a parasitic egg, however, does not necessarily indicate an inability to recognize it, as hosts may sometimes accept the foreign egg even after recognizing it (Moskát and Hauber 2007; Soler et al. 2012, 2017; Zhang et al. 2022). Therefore, distinguishing between egg recognition and egg rejection is essential for understanding host responses to brood parasitism.

Undoubtedly, the ability to visually detect and eject parasitic eggs is crucial for hosts (Guigueno et al. 2014; Li et al. 2020; Soler et al. 2014). The optimal acceptance threshold hypothesis proposes that hosts reject eggs when the perceived difference between parasitic and host egg exceeds a certain threshold (Hanley et al. 2019; Hauber et al. 2006). Previous studies have predominantly examined the magnitude of differentiation between parasitized and host eggs (Hanley et al. 2019; Hauber et al. 2006, 2021), with evidence suggesting that host egg recognition primarily operates through fixed sensory thresholds (Spottiswoode and Stevens 2010). However, hosts may also improve recognition accuracy by increasing inspection effort, thereby facilitating comparisons between eggs. Supporting this idea, studies on great reed warblers *Acrocephalus arundinaceus* have shown that prolonged inspection is associated with faster egg rejection (Požgayová et al. 2011). In contrast, studies on yellow warblers *Setophaga petechia* found that increased inspection did not predict recognition or ejection outcomes (Guigueno and Sealy 2012).

The Daurian redstart (hereafter redstart) is a passerine species exhibiting egg color dimorphism, with females laying either blue or pink eggs (Yang et al. 2016; Zhang, Møller, et al. 2021). This species is a common host of the common cuckoo *Cuculus canorus* , which typically lays light blue parasitic eggs. Redstarts mainly defend against parasitism by rejecting the parasitic egg, although rejection behavior varies markedly among individuals. Overall, roughly half of female redstarts reject the parasitic egg, whereas males do not participate in egg rejection (Zhang, Møller, et al. 2021). Previous studies have shown that egg rejection in this species is influenced by egg mimicry (Zhang, Santema, et al. 2021), parasitism risk (Zhang et al. 2022), and individual behavioral traits (Zhang, Møller, et al. 2021). Nevertheless, much of the observed variation remains unexplained. In this study, we investigated the role of egg‐inspection behavior in host defense. Specifically, we asked whether redstarts adjust inspection behavior when encountering the foreign egg, whether inspection behavior is associated with egg recognition ability, and whether variation in inspection predicts egg ejection.

## 2 Methods

### 2.1 Study Species, Study Site, and General Procedures

Fieldwork was conducted in ShuangYu village, Jilin Province, northeastern China (43°37′19″ N, 126°09′54″ E), during the 2020 and 2021 breeding seasons. The study area covers approximately 300 ha and contains about 240 nest boxes. At this site, Daurian redstarts generally attempt two breeding bouts per season. Egg laying typically begins in mid‐April, with the first peak in late April and a second peak in early June (Zhang, Møller, et al. 2021). Common cuckoos usually arrive at the breeding grounds in mid‐May, when most redstart nests are either at the late incubation stage or already contain nestlings from the first clutch. Consequently, brood‐parasitism pressure varies seasonally, being negligible during the first egg‐laying period and substantially higher during the second (Zhang, Møller, et al. 2021).

During each breeding season, natural nests were located through daily searches, whereas unoccupied nest boxes were inspected weekly. Once a nest became active, regardless of nest type, it was monitored at 1–2 days intervals to document laying date, egg coloration, clutch size, and the occurrence of parasitism.

### 2.2 Artificial Parasitism Experiments

Artificial parasitism experiments were performed during the second egg‐laying period in both 2020 and 2021 by introducing a model cuckoo egg into redstart nests. The egg models were made of clay and painted with acrylic pigments, closely matching the mass, size, and color of natural cuckoo eggs found in redstart nests (Zhang, Møller, et al. 2021; Zhang, Santema, et al. 2021). Experimental parasitism was conducted during the late laying or early incubation stage. To record female behavior, a miniature video camera (HDQ 19, TT cam, China) was installed approximately 1 h before the introduction of the foreign egg. The camera was fixed to the upper interior of the nest box and positioned directly above the nest cup to ensure an unobstructed view. One hour after camera installation, one egg model was added to the focal nest, and recording continued for around 2 h. Nests were subsequently checked once per day for six consecutive days to determine the fate of the foreign egg. The egg was classified as ejected if it disappeared from the nest, and as accepted if it remained present after 6 days. Egg recognition was inferred when females pecked at the foreign egg during the 2‐h observation period, as pecking toward their own eggs was never observed (Zhang et al. 2022). Field observations and video analyses indicated that redstarts remove parasitic eggs by grasping and ejecting them from the nest; therefore, pecking behavior was interpreted as a cue of egg recognition rather than direct ejection in this species. A total of 96 experimental trials were conducted, including 18 nests in 2020 and 78 nests in 2021. Seven nests were predated during the experiment and were therefore excluded from analyses of egg‐ejection propensity. Because artificial nest boxes facilitate optimal camera placement and provide clearer views of female behavior, the vast majority of trials were conducted in nest boxes (93 trials), with only three performed in natural nests.

From video recordings, we quantified both the frequency of egg inspection (i.e., the number of times a female visually inspected the clutch) and the total inspection time (in seconds) during the 1‐h periods before and after the introduction of the egg model. All videos were analyzed by a single observer (Guobin Zheng).

### 2.3 Statistical Analyses

We used two indices to represent individuals' egg inspection, including the frequency of egg inspection and the time of egg inspection.

When females inspected the clutch, it was not possible to precisely determine whether they were inspecting their own eggs or the foreign egg. Therefore, to examine whether changes in inspection behavior were driven by the presence of the foreign egg, we compared inspection frequency and duration between before and after introducing the foreign egg using two generalized linear mixed‐effects models (GLMMs). In each model, inspection frequency or duration was used as the response variable, with treatment (before/after), clutch color (blue/pink), and year as fixed effects, and nest identity as a random effect.

We then examined how egg‐inspection behavior was associated with egg‐rejection outcomes (egg recognition and egg ejection) using GLMMs. In the first model, egg recognition (yes/no) was used as the response variable, with inspection frequency and clutch color (blue or pink) as fixed effects, and year included as a random effect. In the second model, egg recognition was included as the response variable, with inspection duration and clutch color as fixed effects, and year as a random effect. In the third model, egg ejection (accepted vs. ejected) was used as the response variable, with inspection frequency and clutch color as fixed effects, and year as a random effect. In the fourth model, egg ejection was the response variable, with inspection duration and clutch color as fixed effects, and year as a random factor.

For each GLMM, both marginal and conditional coefficients of determination (*R* ^2^) were calculated to quantify model explanatory power following Nakagawa et al. (2017). All statistical analyses were conducted using R version 4.0.3 (R Core Team 2020). Statistical significance was evaluated at *α* = 0.05, and results are reported as mean ± SD.

## 3 Results

**FIGURE 1:**
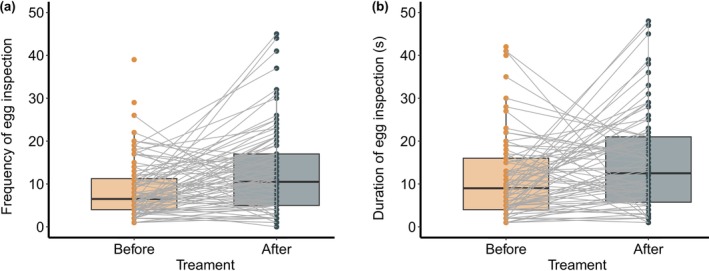
Comparisons of egg‐inspection frequency (a) and duration (b) between before and after the introduction of the foreign egg. Box plots show the median, first, and third quartile, and 1.5 × interquartile range, the gray line indicates how the indices change, and points show raw data.

Daurian redstarts spent more time inspecting the clutch (Table 1; Figure 1a) and inspected it more frequently (Table 1; Figure 1b) after introducing the foreign egg (inspection time and frequency were 12.92 ± 13.41 s and 9.13 ± 8.76 before, and 23.78 ± 28.11 s and 15.01 ± 16.2 after introducing the foreign egg, respectively; *N* = 96). The results indicate that redstarts showed a specific response to the foreign egg, namely, an increased intensity of egg inspection.

**TABLE 1:** Results from GLMMs examining differences in egg‐inspection frequency and duration between before and after introducing the foreign egg.

Dependent variable	Fixed effect	Estimate	SE	*t*	*p*
Inspection frequency	Intercept	2.79	0.16		
	Treatment (before)	0.51	0.04	11.84	< 0.0001
	Clutch color (blue)	0.28	0.13	2.09	0.036
	Year (2021)	−0.02	0.15	−0.15	0.883
Inspection duration	Intercept	2.76	0.17		
	Treatment (before)	0.63	0.04	17.64	< 0.0001
	Clutch color (blue)	0.29	0.14	2.05	0.041
	Year (2021)	0.11	0.15	0.72	0.473

*Note:* For each fixed effect, the reference category is indicated in parentheses.

**FIGURE 2:**
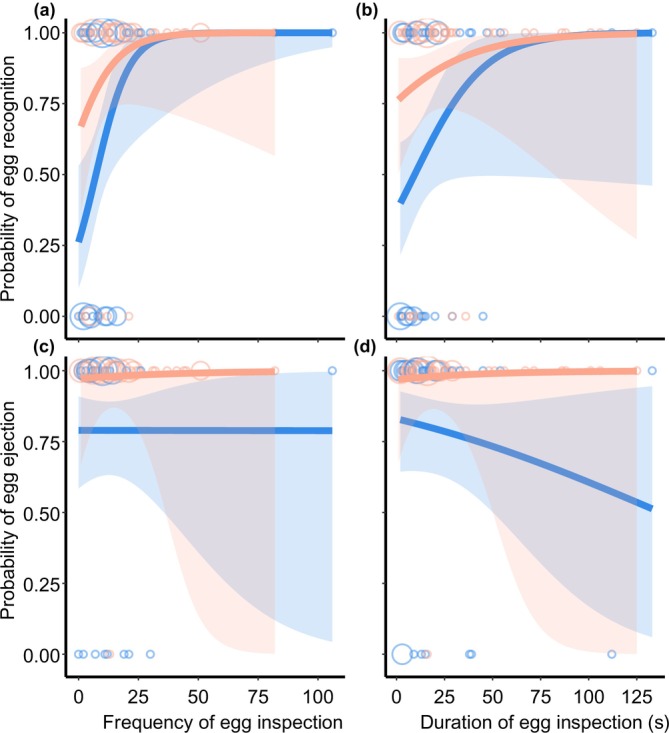
Relationships between egg‐inspection behavior and the probability of egg recognition (a, b) or the probability of egg ejection (c, d). Plot color indicates the clutch color, where blue indicates blue clutch and pink indicates pink clutch. The gray shading in indicates the 95% confidence intervals from GLMMs, dots show the raw data, and dot size represents the number of individuals (*N* = 1–10).

We then examined the relationship between egg inspection and egg‐rejection behavior. Among 96 trials, 70 females (44 laying pink eggs and 26 laying blue eggs) recognized the foreign egg (i.e., pecking at it) during the 2‐h presentation period. In total, 80 females ejected the foreign egg, whereas nine individuals accepted it. Among those that recognized the foreign egg, 22 females ejected it (20 laying pink eggs and two laying blue eggs), whereas six individuals ultimately did not eject it (five laying blue eggs and one laying pink eggs). These results indicate that, after recognizing a parasitic egg, females laying pink eggs were significantly more likely to reject it (Fisher's exact test: *p* < 0.001), whereas females laying blue eggs were more likely to ultimately accept it (Fisher's exact test: *p* = 0.023). Moreover, females that inspected the parasitic egg more frequently and for longer durations were more likely to recognize the foreign egg (Table 2, model 1, 2; Figure 2a,b). However, neither inspection frequency nor inspection duration was significantly associated with the probability of egg ejection (Table 2, models 3, 4; Figure 2c,d).

**TABLE 2:** Results from GLMMs predicting the probability that female redstarts recognized and ejected the foreign egg.

Model	Dependent variable	Fixed effect	Estimate	SE	*z*	*p*	*R*_m_^2^	*R*_c_^2^
1	Egg recognition	Intercept	−0.61	0.74			0.59	0.64
		Inspection frequency	0.13	0.04	2.93	0.003		
		Clutch color (blue)	1.43	0.56	2.54	0.011		
2	Egg recognition	Intercept	−0.13	0.69			0.42	0.48
		Inspection duration	0.05	0.02	2.14	0.032		
		Clutch color (blue)	1.53	0.55	2.79	0.005		
3	Egg ejection	Intercept	1.26	1.16			0.22	0.51
		Inspection frequency	−0.003	0.03	−0.11	0.909		
		Clutch color (blue)	2.47	1.12	2.21	0.027		
4	Egg ejection	Intercept	1.57	1.21			0.24	0.54
		Inspection duration	−0.01	0.01	−1.12	0.262		
		Clutch color (blue)	2.59	1.14	2.27	0.023		

*Note:* *R*_m_^2^ and *R*_c_^2^ indicate marginal and conditional *R* ^2^ values for models. For clutch color, the reference category is blue.

## 4 Discussion

Our results show that Daurian redstarts increased both the frequency and duration of egg inspection following the introduction of a foreign egg. Females that inspected the foreign egg more frequently and for longer durations were more likely to recognize it. Among individuals that recognized the parasitic egg, those laying pink eggs were more likely to reject it, whereas those laying blue eggs were more likely to ultimately accept it. However, egg‐inspection behavior was not associated with their egg‐ejection decisions.

The increase in both inspection duration and frequency suggests that the presence of a foreign egg triggers heightened attention in host females. This behavioral response indicates that an unfamiliar egg in the clutch may be perceived as a potential threat, even when it is not immediately identified as parasitic. Similar patterns have been reported in great reed warblers, in which females increased inspection effort following the introduction of real or artificial cuckoo eggs (Požgayová et al. 2009). The presence of a conspicuously foreign object may, therefore, elevate host vigilance, facilitating subsequent egg discrimination and ejection processes (Feng et al. 2019; Yang et al. 2015). It is also possible that the increase in inspection effort was partly driven by the enlargement of clutch size following experimental manipulation, as no host eggs were removed during the experiment. However, previous studies have shown that hosts are generally insensitive to clutch size variation and that egg recognition is not based on the number of eggs present (Liu et al. 2023; Moksnes and Røskaft 1989). Therefore, the observed behavioral changes are more likely to reflect responses to egg appearance rather than clutch size.

We further found that individuals who inspected the foreign egg more frequently and for longer durations were more likely to recognize it. This result suggests that egg inspection functions as an active anti‐parasitic mechanism that enhances recognition accuracy by allowing hosts to gather more information for comparison. This finding is consistent with results from great reed warblers (Požgayová et al. 2011). Frequent inspection may enable hosts to repeatedly compare the foreign egg with their own eggs, thereby improving discrimination accuracy. This pattern is also consistent with the optimal acceptance threshold hypothesis, which predicts that hosts refine their discrimination decisions on the basis of accumulated sensory information (Hanley et al. 2019; Hauber et al. 2006). Interestingly, hosts laying different egg colors showed distinct responses after recognizing the foreign egg. Females laying pink eggs were more likely to reject the foreign egg, whereas individuals laying blue eggs were more likely to ultimately accept it. This difference reflects variation in egg mimicry: the parasitic egg differs more markedly from pink eggs than from blue eggs, making recognition and ejection easier for pink‐egg layers, whereas blue‐egg layers may require more prolonged assessment. This interpretation is consistent with previous studies showing that non‐mimetic eggs are rejected rapidly, whereas mimetic eggs require longer inspection to avoid recognition errors (Honza et al. 2004; Lotem et al. 1995). Rejecting highly mimetic eggs is cognitively more demanding and often involves longer decision times (Davies et al. 1996; Stokke et al. 2005).

Despite the clear link between inspection behavior and egg recognition, we found no evidence that inspection behavior influenced egg‐ejection decisions. This further supports the notion that egg recognition and egg rejection are behaviorally and cognitively dissociable processes (Ruiz‐Raya and Soler 2017). After recognizing a parasitic egg, hosts may still make conditional decisions on the basis of ecological or individual factors rather than relying solely on recognition. For instance, previous work has shown that redstarts adjust their egg ejection behavior according to perceived parasitism risk (Zhang et al. 2022). Specifically, when the parasitism risk was high, all individuals ejected the foreign egg after recognizing it, whereas half of the individuals chose to accept the recognized foreign egg when there was no parasitism risk (Zhang et al. 2022). In the present study, we found that some individuals recognized the foreign egg but did not eject it in the end. A possible explanation might be the risk of brood parasitism; however, all experiments were conducted during the second egg‐laying period, indicating that all hosts suffered the same level of parasitism risk (Zhang et al. 2022). This suggests that additional factors, such as personality, may play a role in egg‐ejection decisions (Moskát et al. 2014; Zhang, Santema, et al. 2021).

In conclusion, our study shows that Daurian redstarts increase egg‐inspection effort in response to the foreign egg, and that such behavior enhances egg recognition. However, inspection behavior does not predict egg ejection, highlighting a decoupling between recognition and rejection processes.

## Author Contributions

**Guobin Zheng:** data curation (equal), formal analysis (equal), investigation (equal), methodology (equal), visualization (equal), writing – original draft (equal), writing – review and editing (equal). **Jinggang Zhang:** conceptualization (equal), data curation (equal), formal analysis (equal), funding acquisition (equal), investigation (equal), methodology (equal), resources (equal), visualization (equal), writing – original draft (equal), writing – review and editing (equal). **Zixuan Lin:** methodology (equal), writing – review and editing (equal). **Lixing Yang:** methodology (equal), writing – review and editing (equal). **Jianqiang Li:** conceptualization (equal), writing – review and editing (equal). **De Chen:** conceptualization (equal), writing – review and editing (equal). **Donglai Li:** funding acquisition (equal), writing – review and editing (equal). **Wenhong Deng:** conceptualization (equal), funding acquisition (equal), project administration (equal), supervision (equal), writing – review and editing (equal).

## Funding

This study was supported by the National Natural Science Foundation of China (32501383 to J.Z.; 31672297 and 32271559 to W.D.; 31911540468 to D.L.), Basic Scientific Research Projects of Liaoning Provincial Department of Education (LJKZ0093 to D.L.), and the Fundamental Research Funds for the Central Universities (312200502560 to J.Z.).

## Ethics Statement

Our study adheres to the ASAB/ABS guidelines for the treatment of animals in research. The experiments comply with the current laws of China, where they were performed. Fieldwork was carried out with permission from the Yongji Forestry Bureau, Jilin, China. Experimental procedures were conducted under license from the Animal Management Committee at the College of Life Sciences, Beijing Normal University (permit no. CLS‐EAW‐2018‐001).

## Conflicts of Interest

The authors declare no conflicts of interest.

## Supporting information


**Data S1:** ece373595‐sup‐0001‐DataS1.xlsx.

## Data Availability

All data generated or analyzed during this study are included in Data S1.
